# Crossing the line? Reflecting on interdisciplinary boundaries in veterinary science

**DOI:** 10.1002/vro2.70017

**Published:** 2025-08-25

**Authors:** Sebastián Moya Durán, Kate Lamont, Snehan Peshin, Cristóbal Briceño

**Affiliations:** ^1^ School of Veterinary Medicine and Science University of Nottingham Nottingham UK; ^2^ Centre for Epidemiology and Planetary Health, School of Veterinary Medicine Scotland's Rural College Inverness UK; ^3^ Department of Mechanical and Aerospace Engineering University of California Irvine California USA; ^4^ Departamento de Medicina Preventiva Animal, Facultad de Ciencias Veterinarias y Pecuarias Universidad de Chile Santiago Chile

In recent years, veterinary science has undergone a quiet yet significant transformation, marked not only by multidisciplinarity (ie, the combination of different disciplines, each of which provides a different perspective on an issue), but also by a growing openness to interdisciplinarity (ie, the coherent and integrated harmonisation of knowledge, skills and experiences from diverse disciplines).^1^

This interdisciplinary phenomenon has not emerged by chance; it is instead a response to concepts such as One Health, which have challenged us to rethink the boundaries between animals, people and the environment.^2^ In this context, the sustained rise of research at the animal–human–environment interface^3^ is not a trend but a necessity. Interdisciplinarity is also equally valued in applied settings such as public health, biomedical engineering and wildlife conservation, where innovation often arises out of necessity, and crossdisciplinary thinking is strongly oriented towards problem solving.

Yet, amid this transformative enthusiasm, questions arise that demand pause and reflection. For example, where do we draw the line between genuine dialogue and appropriation among disciplines? What at first appears to be a collaboration could instead conceal subtle forms of colonisation. Epistemology—the branch of philosophy that studies knowledge, how we acquire it, what justifies it and what makes it valid or reliable—is relevant here, as it helps us question the assumptions behind an interdisciplinary perspective.

An increasing number of studies incorporate conceptual or methodological tools from other disciplines without adequately involving individuals who are formally (eg, through certified degrees or courses) or informally (eg, through substantial engagement with the literature, mentorship, or progressive learning and practising) trained in those fields.^4^ In such contexts, it would be advisable for researchers to be given space to undertake appropriate training rather than merely carrying out research in the absence of such grounding. Yet, in many cases, universities do not maintain a record of this type of training—an issue that should be made transparent, if not by the institutions themselves, then by the researchers.

To that end, researchers should be encouraged to explicitly disclose their disciplinary background and relevant training when writing up their studies. Doing so would help foster a more reflexive and accountable approach to knowledge production; one that critically engages with positionality and bias to promote rigour and ethical integrity.^5^ Exploring another field is one thing; assuming authority within it without engagement with its foundational logics or without collaborating with domain experts is quite another. The warnings in the literature are clear—adopting external frameworks without sufficient grounding can lead not only to fundamental misunderstandings or mistakes, but also to ethical distortions, undermining the quality, and above all, the legitimacy, of research.^6^

What is at stake is not just the perspective, but the standpoint from which one speaks. For this reason, some philosophers have coined the term ‘epistemic trespassing’^7^ – that figure of the expert who, seduced by the breadth of another field, ventures forth without a map and, more importantly, without humility. Genuine interdisciplinarity demands not only openness, but also a real process of recognition. This means acknowledging that not everything can be understood from the outside, and that some forms of knowledge, skills and experience require presence, attentive listening and, above all, crediting the other not just as a valid interlocutor but as a necessary one.^8^

These tensions become particularly evident when interdisciplinarity involves only two disciplines, for example, veterinary science and social science.^9^ In such cases, one discipline may become crystallised as marginal or even dispensable, as though its contributions could be tacitly subsumed by the other. This dichotomy not only devalues a holistic understanding of the sidelined discipline but also limits frugal innovation (ie, practical, low‐resource solutions adapted to real‐world constraints).

On more than one occasion, the contributions of professionals from these marginalised disciplines have been relegated to a footnote while individuals without relevant training in the discipline assume the role of authorised spokespersons.^10^ This is not merely an epistemic injustice; it is a weakening of research. What foundations are lost when those who could enrich it are silenced? What remains unaddressed when harmonisation is merely performative?

Underlying these dynamics is not merely a matter of recognition, but a power structure that operates quietly within veterinary science. This can undermine disciplines that are community driven, context responsive or oriented towards design, application and evaluation. Although these disciplines may offer pragmatic but nontraditional pathways to progress, they risk being subjected to an excessively hierarchical logic, in which certain disciplines are regarded as more legitimate than others.^11^ This dominant epistemology not only determines which epistemic foundations circulate but also establishes a kind of disciplinary filter.

Epistemological conflicts are long standing. However, with the current trend towards greater interdisciplinarity, a genuine understanding of the fundamental differences between disciplines appears to have been lost. Beyond acknowledging their differing perspectives, awareness and respect for the respective research methodologies of each discipline are also vital elements of interdisciplinary research. The consequences of not incorporating these elements are both tangible and far reaching.

One such consequence is the previously discussed lack of appropriately trained individuals within interdisciplinary projects, thereby sustaining hegemonic frameworks and rigid perspectives where any conceptual or methodological deviation is seen mainly as a threat rather than a contribution.^12^, ^13^ In such a context, those who appropriately cross disciplinary boundaries—equipped with relevant training—often do so at an even greater disadvantage, constantly being required to justify their legitimacy in front of those who claim to grasp the foundations of such disciplines without ever acknowledging their own lack of disciplinary grounding. Would it be possible to imagine someone conducting a clinical examination of a sick animal, including diagnostic testing, without appropriate veterinary training or supervision? Probably not. So why is it that, in a research setting, one discipline can navigate into others without sufficient grounding?

**FIGURE 1:**
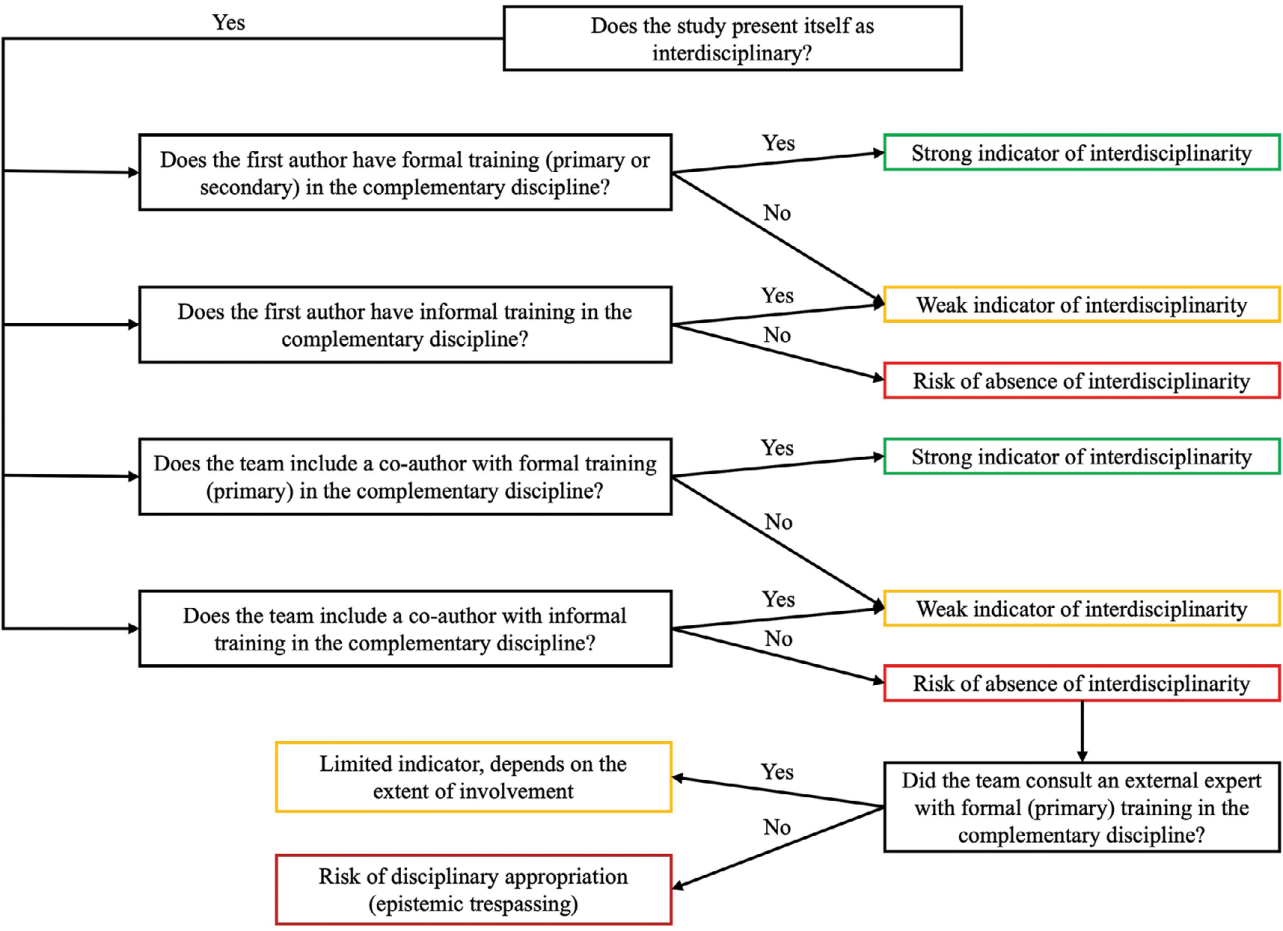
Decision tree for orienting interdisciplinary assessment in research authorship. Green boxes indicate strong evidence of interdisciplinarity, based on formal training held by the first author and/or at least one co‐author. Yellow boxes represent weak interdisciplinarity, based on informal training held by the first author and/or at least one co‐author. Red boxes signal a clear risk of absence of interdisciplinarity and, in more critical cases, the risk of disciplinary appropriation (ie, epistemic trespassing), where neither formal nor informal training is evident.

So, how do we move forward? How can we prevent interdisciplinarity from becoming just another hollow slogan? One possible path lies in developing frameworks that allow us to discern when collaboration is genuine, and when it is merely academic window dressing. In this spirit, we propose a decision tree for orienting interdisciplinary assessment (Figure 1). This tool is intended mainly for researchers, but also for reviewers, editors and evaluators (eg, for academic positions or project proposals), and can be applied both during the early phases of research and at later stages such as manuscript review, interviews or project proposal evaluation.

Within this framework, studies that explicitly incorporate at least one author with formal training in the complementary discipline could be considered to be on the right path (Figure 1). Even those that do not may still offer valuable contributions, provided they acknowledge their limitations and clearly frame their work as a disciplinary incursion. While the proposed framework promotes transparency regarding interdisciplinary credentials, the context in which studies are conducted must also be taken into account, as, in some circumstances, service users or other stakeholders may be considered as contributing to disciplinary expertise.^1^ The purpose of this framework is not to offer a definitive assessment, but rather to open up a space for reflection on interdisciplinary practice, recognising that this may vary substantially across fields. Ultimately, we encourage a gesture of humility.

However, we must avoid tendencies that define interdisciplinarity through any form of segregation. Instead, we should foster it through genuine and responsible engagement, as interdisciplinarity goes beyond merely combining disciplines—it integrates them to address complex problems. We must build spaces where each discipline can speak from its own roots, without being subsumed. In this endeavour, the veterinary scientific community has both an opportunity and a responsibility to rethink itself and to explore forms of collaboration that do not reproduce the hierarchies of the past.^14^

Ignoring these questions is not a neutral act, as interdisciplinarity must be understood as a political act—one of resistance to reduction and a commitment to just and plural modes of understanding. Perhaps that is precisely what is needed today; a science that, rather than retreating into its certainties, is sufficiently brave and humble to navigate interdisciplinary intricacy.
